# The pattern of alternative splicing in lung adenocarcinoma shows novel events correlated with tumorigenesis and immune microenvironment

**DOI:** 10.1186/s12890-021-01776-0

**Published:** 2021-12-06

**Authors:** Gongjun Wang, Weiwei Qi, Liwei Shen, Shasha Wang, Ruoxi Xiao, Wenqian Li, Yuqi Zhang, Xiaoqian Bian, Libin Sun, Wensheng Qiu

**Affiliations:** 1 Department of Oncology, The Affiliated Hospital of Qingdao University, Qingdao, Shandong China; 2 Department of Oncology, Women and Children’s Hospital, Qingdao University, Qingdao, Shandong China; 3 Department of Medcine, Qingdao University, Qingdao, China

**Keywords:** LUAD, Prognosis, Alternative splicing, Immune, Tumor microenvironment

## Abstract

Lung adenocarcinoma (LUAD) is the leading cause of cancer deaths worldwide due to the lack of early diagnostic markers and specific drugs. Previous studies have shown the association of LUAD growth with aberrant alternative splicing (AS). Herein, clinical data of 535 tumor tissues and 59 normal tissues were extracted from The Cancer Genome Atlas (TCGA) database. Each sample was analyzed using the ESTIMATE algorithm; a comparison between higher and lower score groups (stromal or immune) was made to determine the overall- and progression-free survival-related differentially expressed AS (DEAS) events. We then performed unsupervised clustering of these DEASs, followed by determining their relationship with survival rate, immune cells, and the tumor microenvironment (TME). Next, two prognostic signatures were developed using bioinformatics tools to explore the prognosis of cases with LUAD. Five OS- and six PFS-associated DEAS events were implemented to establish a prognostic risk score model. When compared to the high-risk group (HRG), the PFS and OS of the low-risk group (LRG) were found to be considerable. Additionally, a better prognosis was found considerably associated with the ESTIMATE score of the patients as well as immune cells infiltration. Our analysis of AS events in LUAD not only helps to clarify the tumorigenesis mechanism of AS but also provides ideas for revealing potential prognostic biomarkers and therapeutic targets.

**Supplementary Information:**

The online version contains supplementary material available at 10.1186/s12890-021-01776-0.

## Introduction

lung carcinoma (LC) is known as one of the most prevalent forms of carcinoma and is the underlying reason for cancer mortality in China [1]. In the US, it possesses a 5-year survival rate of 14% [2]. Delayed diagnosis, particularly after metastasis, is one of the major causes for its poor prognosis for LC [3]. Approximately 85% of all cases of LC have non-small cell LC (NSCLC), including varying histological types, such as LUAD, squamous cell carcinoma, and large cell LC, with LUAD being the most common type of NSCLC [4]. Presently, there are no reliable biomarkers available for predicting prognosis in patients suffering from LUAD, which, in turn, requires the need for the identification of suitable prognostic markers.

Alternative splicing (AS) is a critical post-transcriptional process that involves the formation of protein isoforms with varied structural and functional characteristics. AS has a considerable contribution in the modification of > 95% of human genes and is thus, commonly used to explore proteome diversity and cellular complexity [5–7]. Numerous investigations have revealed the association between AS abnormal expression and the pathogenesis of several cancers, including LUAD [8–11]. The splice factor SRSF1 has been shown to modulate PTPMT1 alternative splicing to regulate LC cell radioresistance [12]. Also, whole-genome analysis of AS events in LC has resulted in the identification of several candidate splicing factors, which might act as therapeutic targets of LUAD, and help in disease prognosis of patients via the construction of gene signatures [13, 14], demonstrating the role of AS in LC.

AS is directly linked with tumorigenesis and critically involved in the formation of TME, which includes tumor cells, tumor-associated fibroblasts, immune/inflammatory cells, microvessels, stromal tissues, and many cytokines and chemokines [15–19]. The prognosis of cancer patients is known to be directly related to the immune cell count in the TME, which can act as a useful prognostic marker [20–22]. However, except for some preliminary studies on LC-related AS events [13, 14] and immunological microenvironment [23, 24], there is a lack of sufficient data on the immunological relevance of AS events.

Here, we integrated TME and AS events to conduct a first-of-its-kind analysis of prognostic variables in patients with LUAD. Firstly, the immune and stromal scores of patients were procured with LUAD through exploring public databanks and applying the ESTIMATE algorithm. Next, the Kaplan–Meier (K–M) plots were generated by using the obtained data to investigate prognostic variations between higher and lower score groups (stromal or immune). Subsequently, we searched for DEAS through comparative analysis of AS events in a higher and lower score group (stromal or immune). Then, we created two OS- and PFS-based prognostic signatures and determined that prognostic indicators were independent of other pathophysiological markers. Finally, we discerned distinct AS-based LUAD clusters. We studied the relationship between AS-based clusters and the pathophysiological factors and other immunological characteristics, allowing an improved understanding of the prognosis of patients with LUAD.

## Methods

### Collection of data according to TCGA

We extracted clinical data regarding LUAD tissues and normal lung tissues from TCGA (https://www.cancer.gov/) database and regarding the AS events and the related values of percent-splice-in (PSI) from the TCGA spliceseq databank. The AS events were quantified and analyzed using these PSI values (0–1). To confirm the consistency of underlined AS events, we used samples (%) with PSI values larger than 75%. Finally, the study was done on 490 patients with LUAD.

### Analyzing the link between stromal- or immune-scores and prognosis of LUAD patients

To assess the impact of the microenvironment on tumor cells regarding the evolution of genomic changes, the immune- and stromal-scores for the outcomes of the expression of mRNA were evaluated by using the ESTIMATE algorithm in the R program [25]. The ESTIMATE algorithm provided these scores via ssGSEA. For each sample, the expression pattern was rank-ordered and normalized. After that, a statistic was calculated by integrating the difference between the empirical cumulative distribution function, which is similar to the one used in gene set-enrichment analysis but based on absolute expression rather than differential expression.

### Measurement and prognostic significance of immune- and stromal-scores

The ESTIMATE algorithm was employed for the evaluation of the immune- or stromal-scores, and the R-script was obtained from the website (https://sourceforge.net/projects/estimateproject/). These values were utilized to categorize the patients into higher and lower score groups (immune or stromal). Then, K–M survival plots were created to determine the prognostic significance of each group.

### Screening of DEASs on the basis of immune and stromal scores

To investigate the differences in prognosis between the higher and lower score groups (immune/stromal), we analyzed differential expression with PSI values of AS events. We set a restricted requirement of |logfc| > 0 and FDR/adjusted P < 0.05, corresponding to the elevated and decreased expression of AS events, accordingly, due to the low PSI values of several AS events [26]. The plots of heatmaps and volcano were created via the pheatmap program and ggplot2 in the R computer program, accordingly. Consequently, the intersecting AS events were chosen as DEASs and then further evaluated using a Venn diagram. Furthermore, to display the intersections between the seven types of DEASs in LUAD, the plot of UpSet was constructed using the package of UpsetR in R [27].

### Functional enrichment analysis

Following that, an enrichment analysis of the associated parent genes was conducted. Metascape was employed to evaluate Kyoto Encyclopedia of Genes and Genomes (KEGG) pathways (www.metascape.org) and Gene Ontology (GO) analyses. The data obtained from KEGG and GO analyses were used to indicate the first 20 key pathways, if possible.

### Evaluation of consensus clustering and immune microenvironment (IME)

The cohort of TCGA LUAD was categorized using hierarchical consensus clustering. Patients were grouped into multiple clusters using the ConsensusClusterPlus program, which was utilized to cluster in an unbiased and unsupervised behavior [28]. The Elbow approach and the Gap statistic were used to confirm the ideal number of clusters, resulting in a reliable categorization. The Wilcoxon rank-sum test, often known as the K-W test, was performed to compare immune cells and the TME in the three groups.

### Development of DEASs based on prognostic model

To determine the predictive relevance of DEASs in LUAD patients, researchers used univariate Cox-regression analysis (CRA) to discover survival-correlated DEASs. Following that, the regression of least absolute shrinkage and selection operator (LASSO) was implemented to exclude potential predictors with nonzero coefficients, minimizing model overfitting and obtaining the simplest (smallest parameter) model possible [29]. Based on negative log-likelihood statistics and Akaike Information Criterion, a multivariate-CRA was performed to thoroughly assess how each DEAS contributed to prognosis, verifying DEASs participated in the final prognostic signature. Using the findings of the multivariate-CRA and the PSI values, we estimated the risk scores of individuals with LUAD by the given formula:

$${\text{Risk}}\,{\text{Score }} = \mathop \sum \limits_{i = 0}^{n} \beta i{*}Gi$$

$\beta i$ is the coefficient of gene $i$ in the multivariate Cox analysis; $Gi$ is the expression value of gene $i$; and $n$ is the number of genes in the signature.

On the basis of their score of median risk, cases with LUAD were separated into LRG and HRG, and K–M survival curves were formed to indicate the varied prognoses. We also created ROC curves for 1, 2, and 3 years to show the predicted signatures' percipience.

### Development of nomograms integrating DEAS signatures and clinical variables

Clinical variables, such as age, gender, TNM stage, and American Joint Committee on Cancer (AJCC) stage were obtained from the cBioPortal database [30]. For individuals with LUAD, we used a combination of signatures and clinical factors to perform univariate CA. For the multivariate CRA, factors with a P < 0.05 were chosen as independent prognostic variables. The “rms” program in R was then used to create two prognostic nomograms based on the independent prognostic parameters to predict OS and PFS in LUAD patients. To distinguish between the two nomograms, the C-index was used, followed by the construction of calibration curves to appraise the concordance between actual and nomogram-predicted results.

### Gene set variation analysis (GSVA) between HRG and LRG

The “GSVA” R package was utilized to carry out the GSVA enrichment analysis to evaluate variations in the activation of the biological cascade between the HRG and LRG [31]. An adjusted *P*-value < 0.05 was regarded as statistically considerable.

#### Building of potential SF–AS regulatory network

The SpliceAid2 databank (https://www.introni.it/splicing.html) was employed to obtain splicing factor (SF) data [32, 33]. The analysis of Pearson correlation was carried out for evaluation of the link between the values of SFs and PSI in CAAS events. An association was found when the absolute value of the correlation coefficient was > 0.5 and the P-value was < 0.001. Cytoscape (version3.8.2) was used to create the correlation plot.

#### TIMER analysis

Tumor IMmune Estimation Resource (TIMER, available at http://cistrome.org/TIMER) [34] was used to investigate the correlation between the SOD2 gene expression and immune cells infiltration in LUAD samples from The Cancer Genome Atlas (TCGA). In addition, its SCNA module can compare the levels of tumor infiltration among tumors with different somatic copy numbers alterations of the SOD2 gene.

#### Statistical analysis

The R computer program (ver. 4.1.0) was employed for statistical evaluations. *P* < 0.05 (two-sided) was regarded as statistically considerable.

## Results

### The link between immune- and stromal-scores and the rate of prognosis in LUAD patients

**Fig. 1:**
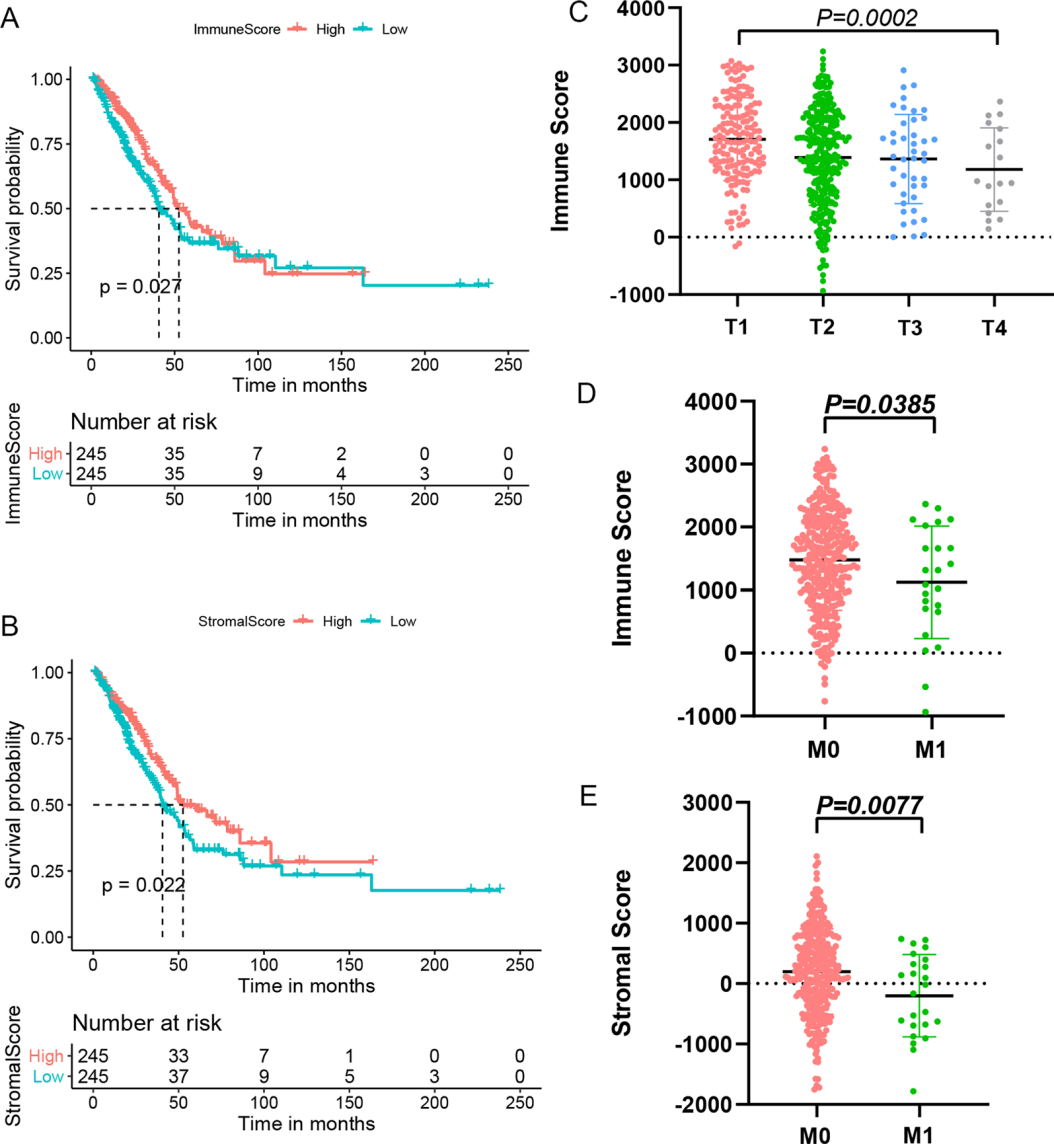
The relationship between stromal/immune scores and prognosis of LUAD patients. **A** The K–M survival curves for OS of high and low immune scores groups. **B** The K–M survival curves for OS of high and low stromal scores groups. **C** Comparison of immune scores between T stages. **D** Comparison of immune scores between M stages. **E** Comparison of stromal scores between M stages

The flow chart of this research is shown in Additional file 1: Fig. 1. The transcriptome outcomes of 490 LUAD cases were taken from the TCGA databank, and the ESTIMATE algorithm was employed to identify their scores (immune/stromal). Then we separated the patients with LUAD into higher and lower score groups (stromal/immune) and examined the variations in prognosis between the two groups using the mean of stromal and immunological scores. Based on the data obtained from K–M curves, lower scores (immune and stromal) were substantially linked with the lower OS in cases with LUAD (Fig. 1A, B). Although no considerable link was detected between immunological and stromal scores with PFS in patients with LUAD (Additional file 2: Fig. 2A, 2B). In addition, the differences in TNM staging between the high and low groups were also compared, and it was found that the T and M stages were statistically significant between the high and low immune groups (Fig. 1C, D), and the M staging was statistically significant between the high and low stromal groups (Fig. 1E).

**Fig. 2:**
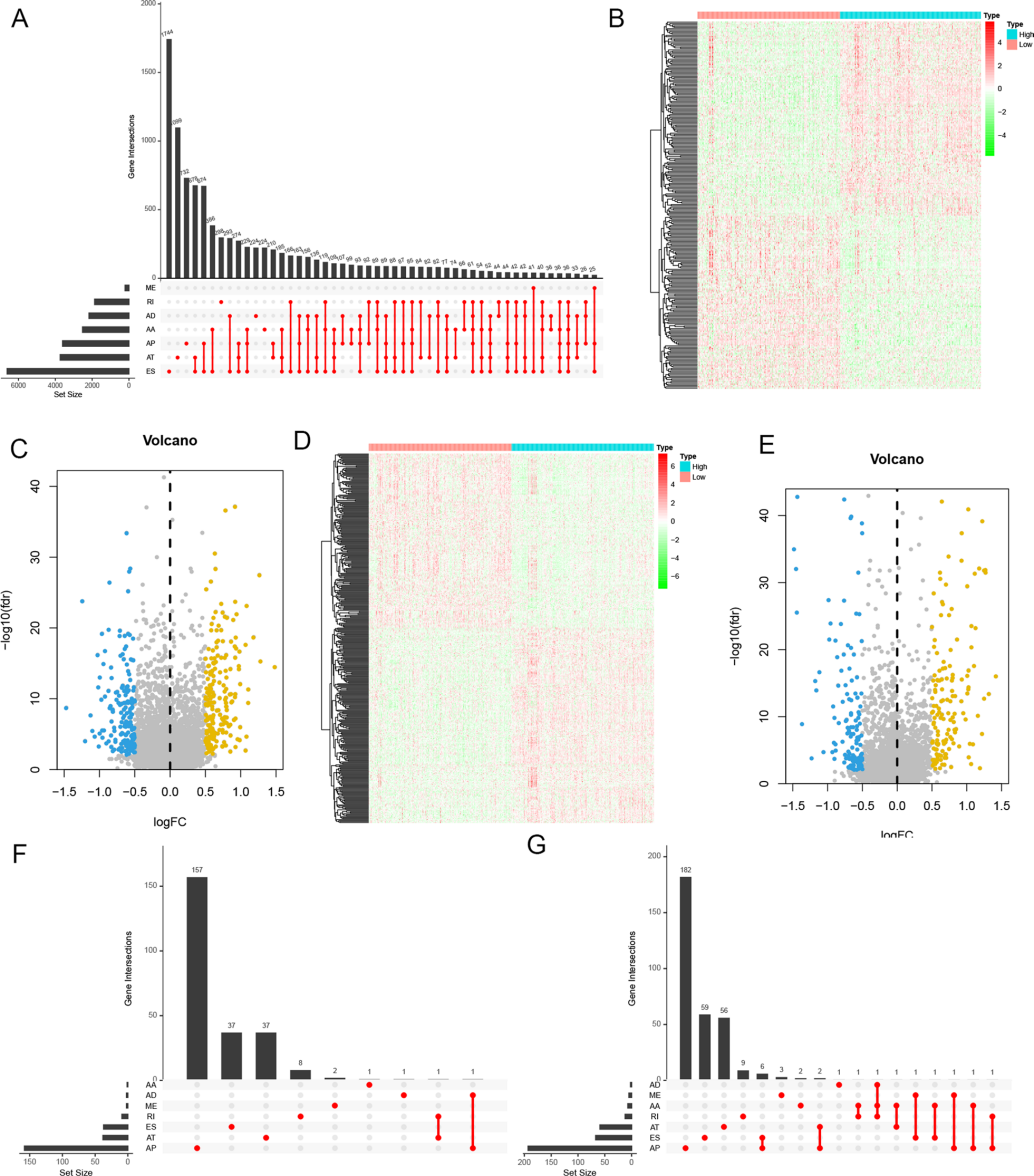
DEAS events between high and low stromal/immune scores groups. **A** The UpSet plot of intersections between AS events and the corresponding gene intersections. The heatmaps (**B**, **D**) and volcano plots (**C**, **E**) of DEAS events between the high and low stromal/immune scores groups. **F**, **G** The UpSet plots of DEAS events and the corresponding gene intersections of the immune group and the stromal group

### DEAS events between higher and lower score groups (immune/stromal)

**Fig. 3:**
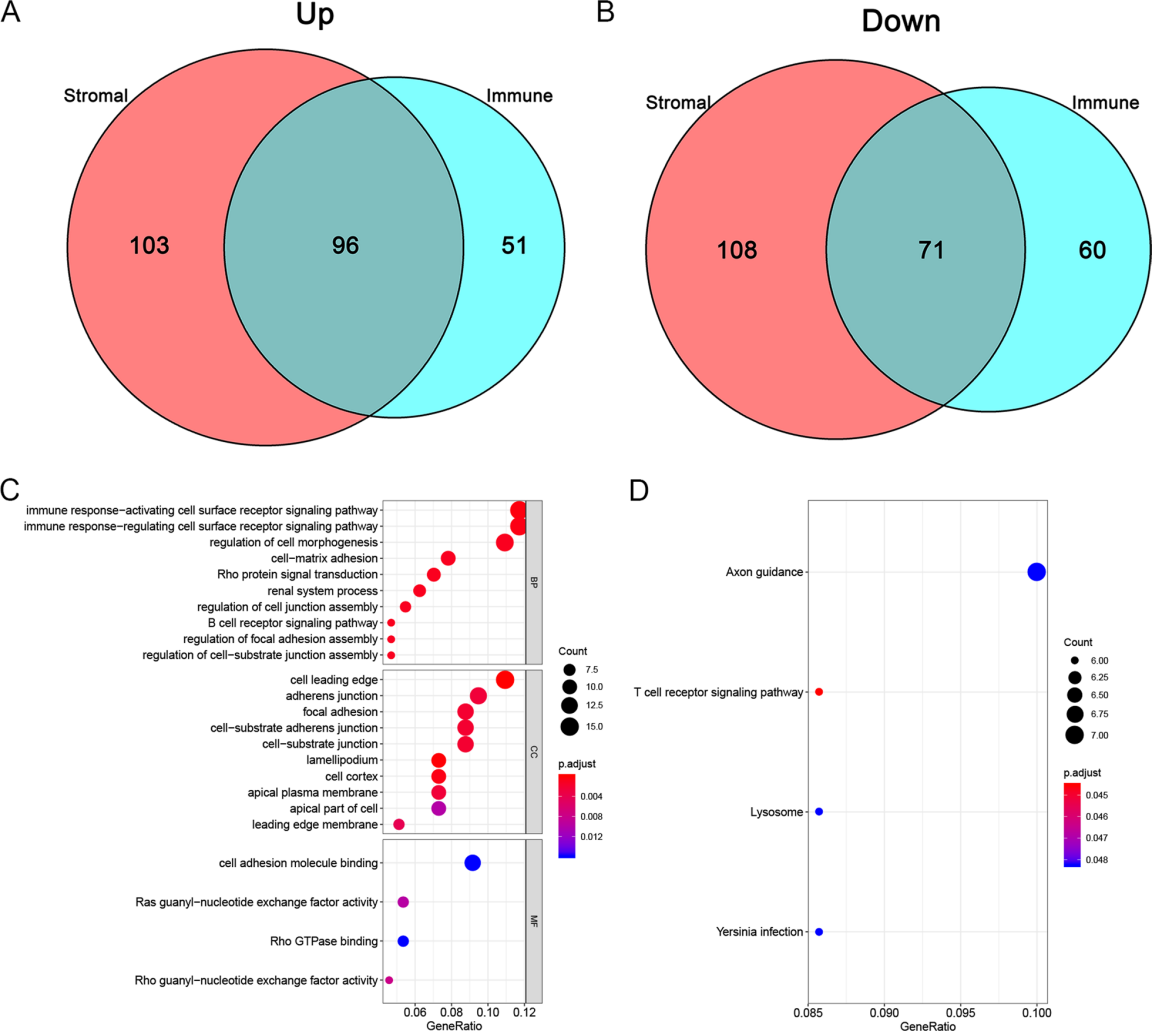
Intersection DEAS events and GO and KEGG pathway enrichment analysis. The up-regulated (**A**) or down-regulated (**B**) DEAS events in both stromal score and immune scores groups by Venn diagram. **C** GO analysis. **D** KEGG pathway enrichment analysis

RNA-Seq data was utilized to create integrated AS event profiling, which led to 43,948 AS events being identified. To guarantee high stringency, the AS events were evaluated using several filters (Standard Deviation ≥ 0.01, average PSI value ≥ 0.05). As a result, 30,569 AS events were discovered. The AS events in LUAD patients were then sorted, and it has been revealed that ES (Exon Skip) was the most common AS event, succeeded by AT (Alternate Terminator), and AP (alternate promoter). The intersections between AS events and the accompanying gene intersections were visualized using the UpSet plot (Fig. 2A). The DEAS events were then evaluated by the comparison of higher and lower score groups (immune or stromal), which were presented using heatmaps (Fig. 2B, D) and volcano plots (Fig. 2C, E). Thus, in the immune groups, 147 DEAS events (Additional file 3: file 1) were highly expressed while the expression of 131 DEAS events (Additional file 4: file 2) was decreased. In the stromal groups, 199 DEAS events (Additional file 5: file 3) were highly expressed while the expression of 179 DEAS events (Additional file 6: file 4)was decreased in the underlined group. The UpSet plots of DEAS events and the related gene intersections of the immune as well as stromal groups are depicted in Fig. 2F, G, respectively. The Venn diagram program was employed to identify 96 up-regulated DEAS events and 71 down-regulated DEAS events shared in the immune groups and the stromal groups (Fig. 3A, B). The upset plot corresponding to the intersection DEAS events was also shown in Additional file 2: Fig. 2C.

The biological events and pathways were then evaluated by the GO and KEGG pathway analysis on the parent genes of DEAS events (Fig. 3C, D). In the BP category, the most enriched pathways were immune response-activating cell surface receptor cascade of signaling and immune response-regulating cell surface receptor cascade of signaling; in the CC category, the most enriched GO terms were adherens junction and cell leading edge; in the MF category, cell adhesion molecule binding and Ras guanyl-nucleotide binding were the primary functions of these genes. For the KEGG pathway enrichment study, the major pathways were Axon guidance and T cell receptor signaling. The enrichment analysis results show that the parental genes of these AS events are closely related to the immune response.

### AS-based clusters are considerably linked with prognosis, and also molecular, and immune characteristics

**Fig. 4:**
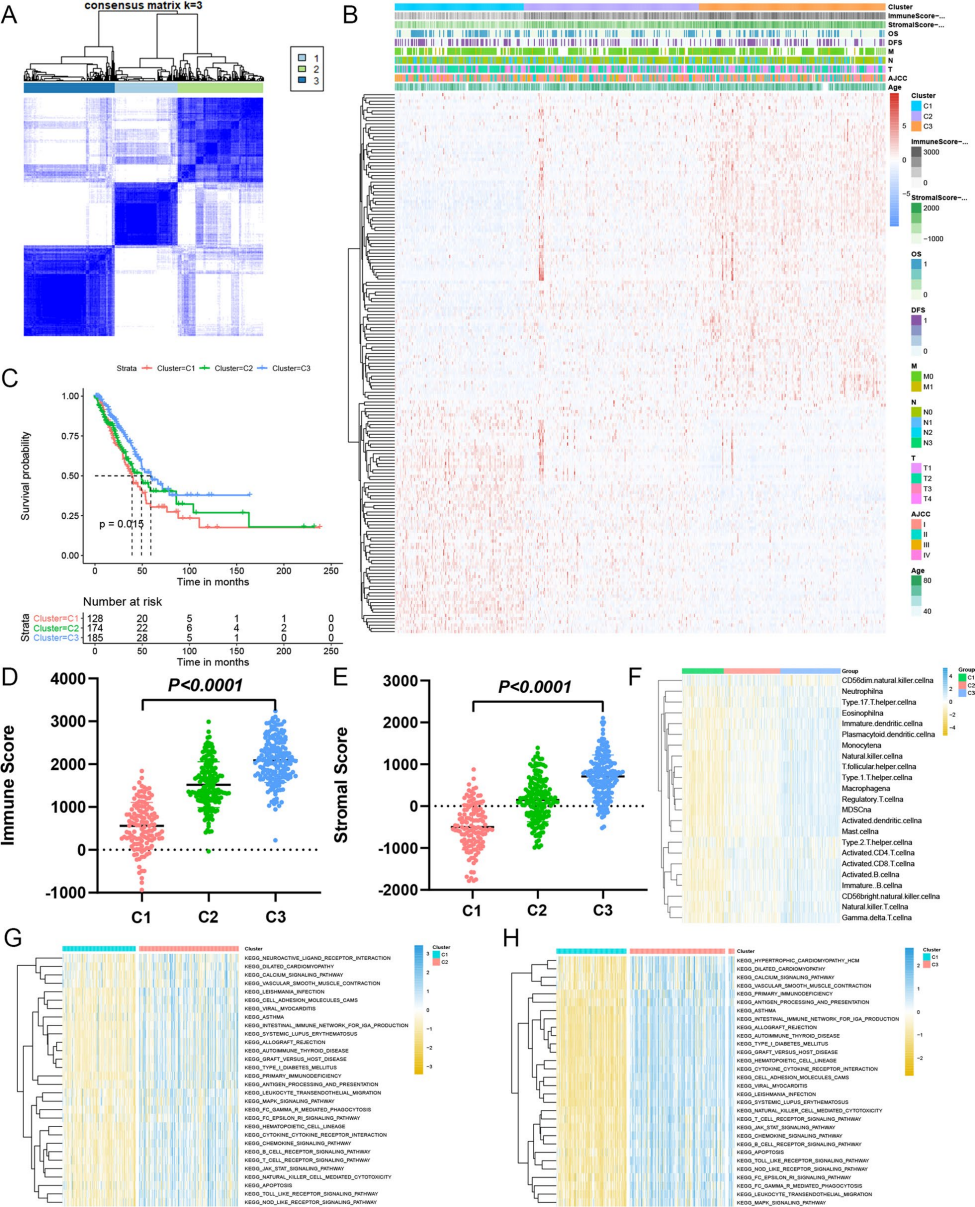
The immune microenvironment was closely related to the prognosis of LUAD patients. **A** TCGA LUAD cohort was clustered into three subgroups by unsupervised cluster analysis. **B** The heatmap of clusters and clinical parameters. **C** K–M survival curves for OS of three clusters. **D**, **E** The comparison of immune scores and stromal scores between three clusters. **F** The comparison of immune cell infiltration between three clusters. **G**, **H** GSVA enrichment analysis showing the activation states of biological pathways in distinct clusters (C1–C2, C1–C3)

The AS profiling revealed that the AS events among LUAD patients were incredibly heterogeneous. This finding makes us wonder whether we can predict the clinical outcome of each patient through the analysis of changes in the expression of AS events. Furthermore, unsupervised consensus analysis was carried out to explore whether AS had any discernible patterns (Fig. 4A). The cluster of each LUAD patient could be seen in Additional file 7: file 5. We found three distinct AS-based molecular clusters based on the consensus matrix heatmap: C1 (n = 128, 26.3%), C2 (n = 174, 35.7%), and C3 (n = 185, 38.0%). To better understand the clinical effects of the evaluated AS clusters, we looked at the correlations between clusters and clinicopathological features, as depicted in Fig. 4B. We could observe that LUAD patients in the three clusters showed different immune and stromal scores. Furthermore, the link of K–M analysis of cluster with prognosis yielded distinct survival patterns. Notably, C3 with the highest immune and stromal scores was linked to a positive OS, whereas C1 and C2 with relatively low immune and stromal scores were related to a negative OS (Fig. 4C). DFS, on the other hand, did not differ considerably among the clusters (Additional file 2: Fig. 2D).

In addition, differences in the immunological microenvironment amongst AS clusters were investigated. The scores (immune and stromal) were determined using the ESTIMATE method to measure the existence of immune and stromal cell infiltration in samples of the tumor. We found that the immune and stromal scores were all highest in C3 (Fig. 4D, E). Furthermore, using ssGSEA, a heatmap was created to examine the relative abundances of the 23 immune infiltrating cell subpopulations among different AS clusters (Fig. 4F). Higher scores (immune and stromal) were related to the C3 cluster, while lower scores (immune and stromal) were connected with the C1 and C2 clusters. The biological behaviors among these different AS patterns were then investigated using a GSVA enrichment analysis. B cell receptor signaling pathway, cytokine-cytokine receptor interaction, natural killer cell mediated cytotoxicity, leukocyte transendothelial migration, T cell receptor signaling pathway and Toll like receptor signaling pathways were among the enrichment cascades presented by the C2 and C3, as depicted in Fig. 4G, H. The results show that patients with LUAD have a better prognosis when their immunological and stromal scores are greater.

### Establishment and assessment of the prognostic signature for LUAD patients

**Fig. 5:**
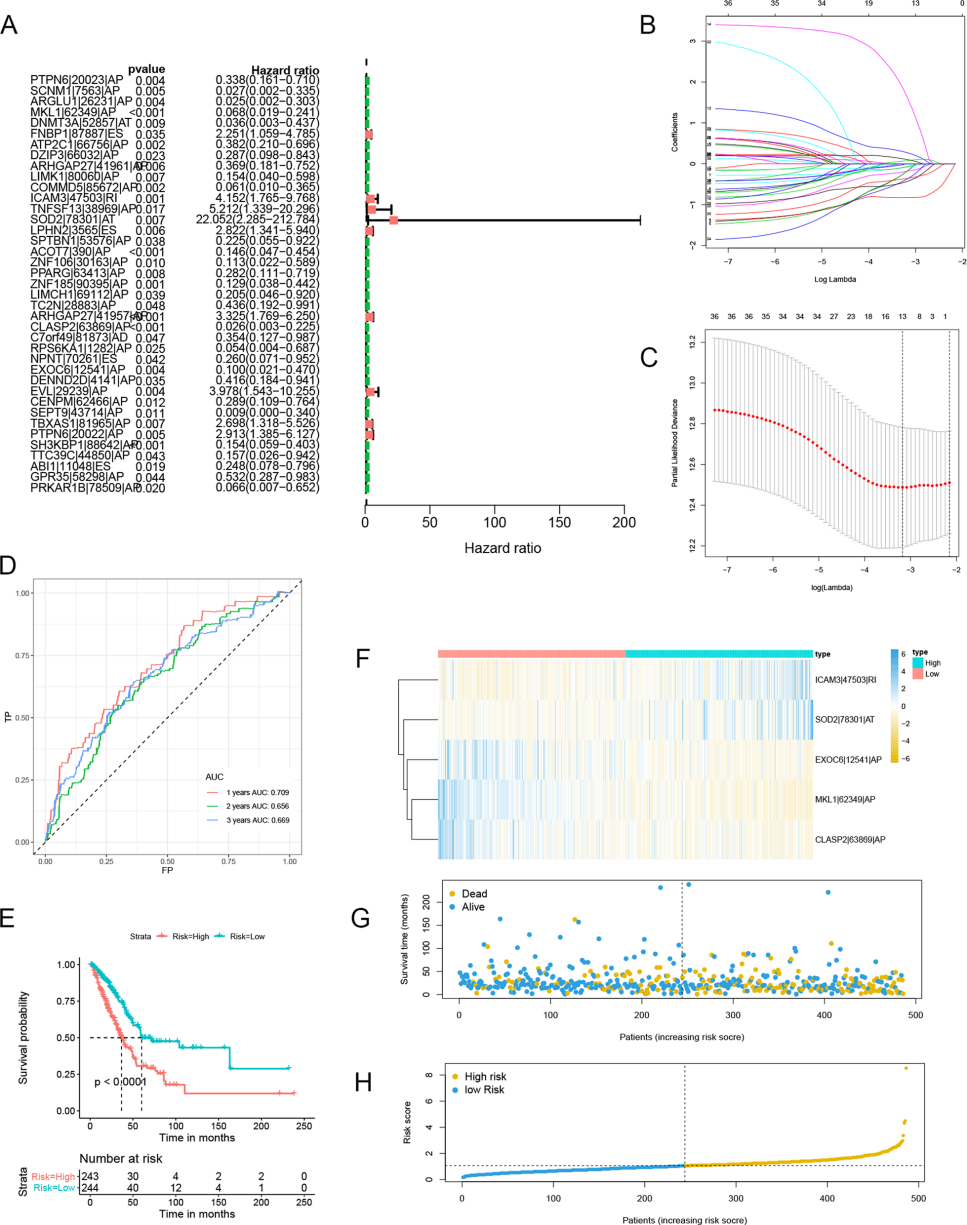
Establishment of a 5-DEAS-based OS signature. **A** The forest plot of the univariate Cox analyses. **B** LASSO coefficient profiles of the candidate survival-related DEASs. A coefficient profile plot was produced against the log λ sequence related to OS. **C** Dotted vertical lines were drawn at the optimal values using the minimum criteria related to OS. **D** ROC curve analysis to evaluate the sensitivity and specificity of the gene signature. **E** K–M survival curve to test the predictive effect of the gene signature. **F** DEAS expression levels of the high-risk and low-risk groups with the OS signatures. **G** OS scatter plots for LUAD patients. **H** Risk score distribution of patients with the OS signatures

**Fig. 6:**
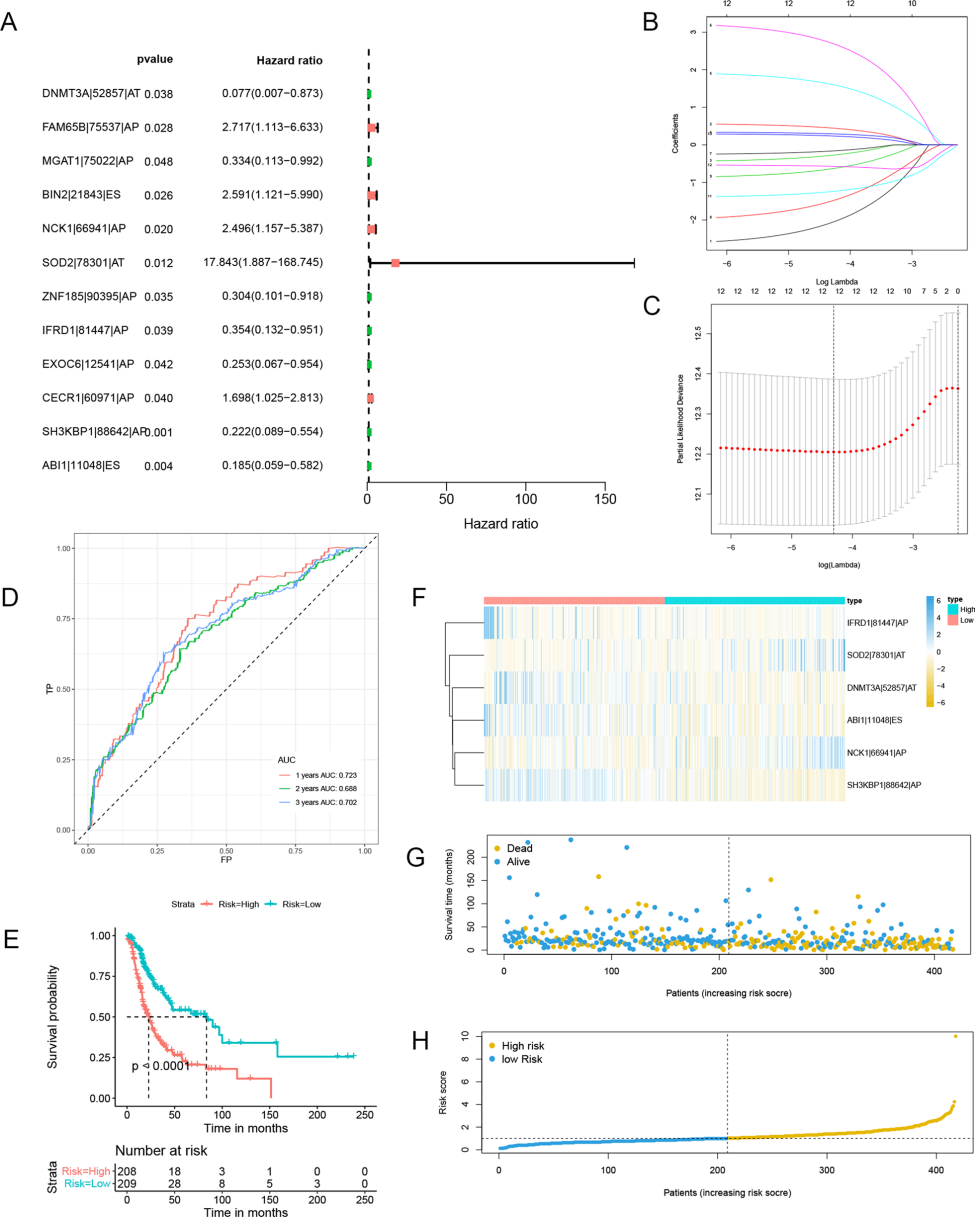
Establishment of a 6-DEAS-based PFS signature. **A** The forest plot of the univariate Cox analyses. **B** LASSO coefficient profiles of the candidate survival-related DEASs. A coefficient profile plot was produced against the log λ sequence related to PFS. **C** Dotted vertical lines were drawn at the optimal values using the minimum criteria related to PFS. **D** ROC curve analysis to evaluate the sensitivity and specificity of the gene signature. **E** K–M survival curve to test the predictive effect of the gene signature. **F** DEAS expression levels of the high-risk and low-risk groups with the PFS signatures. **G** PFS scatter plots for LUAD patients. **H** Risk score distribution of patients with the PFS signatures

Biomarkers for early illness identification and potential therapeutic targets are still a hot topic in medicine. Previous research has found that abnormal AS events in the initial phases of cancer and are used as prognostic markers in a variety of cancers [35, 36]. We generated signatures based on the DEASs to extract the underlying predictive value of individual DEASs, followed by a univariate CRA. The findings demonstrated that in LUAD patients, 39 and 12 intersecting AS events were strongly linked with OS and PFS, accordingly (Fig. 5A, 6A). To avoid model overfitting, the LASSO regression was adopted to select the optimal OS-(Fig. 5B, C) and PFS-(Fig. 6B, C) related DEASs to construct the prediction models. After performing multivariate CRA, 11 DEASs were used to create two prognostic signatures, *i.e.,* 5 DEASs for the OS signature, 6 DEASs for the PFS signature, and one overlapping DEAS. We estimated each LUAD patient's risk score using the formula in the methods, and on the basis of the median of the risk score, we separated the cases into two groups: HRG and LRG. The AUCs of the OS signature to predict 1, 2, and 3 years OS were 0.709, 0.656, and 0.669, accordingly, according to time-dependent ROC curves (Fig. 5D). Patients in the HRG had a lower OS than those in the LRG, according to the K–M curves (Fig. 5E).

The PFS signature's time-dependent ROC curves were also developed in the same way. A median was used to determine the risk score cutoff, and 208 and 209 patients were categorized into two groups: HRG and LRG, respectively. The signature had AUCs of 0.723, 0.688, and 0.702 for predicting PFS after 1, 2, and 3 years, accordingly (Fig. 6D). The PFS was better in the patients with LR scores (Fig. 6E). The underlined data revealed that both signatures were considered to predict the survival rate of LUAD patients. Additionally, heatmaps (Fig. 5F, 6F), survival status plots (Fig. 5G, 6G), and risk score plots (Fig. 5H, 6H) were created to clearly illustrate variations in prognosis and AS patterns.

### Construction of nomograms based on DEAS signature as well as clinical parameters.

To enhance the clinical use of the underlined prognostic signatures, two complete nomograms integrating independent clinical characteristics were created. To investigate independent OS and PFS prognostic variables, we first used univariate and multivariate CRA. The obtained data revealed that Risk scores, T, and N were three independent variables associated with OS and PFS (Additional file 8: file 6, Additional file 9: file 7). These findings showed that both DEASs-based signatures might be employed to predict the prognosis of LUAD cases independently.

**Fig. 7:**
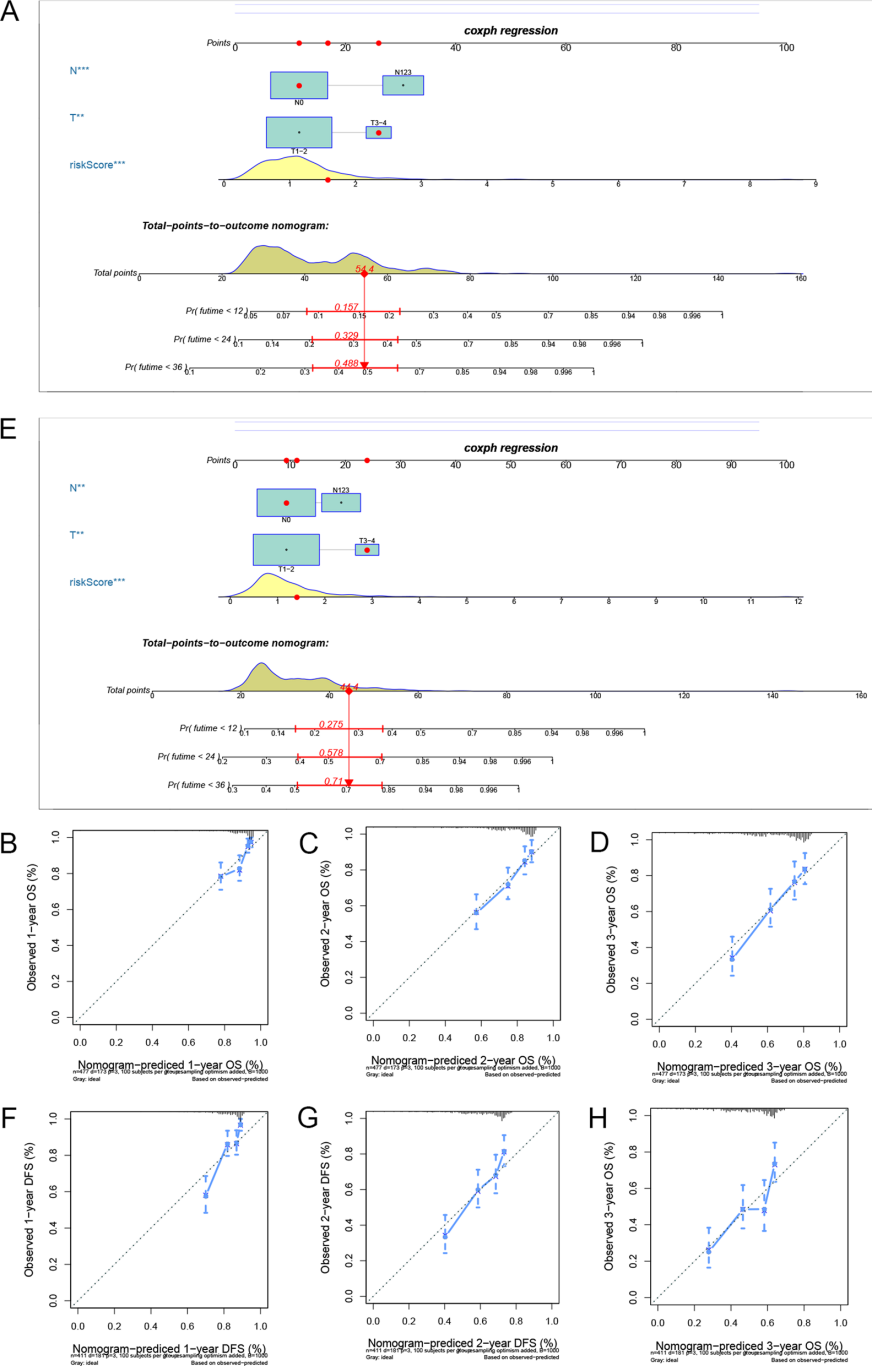
Development of two nomograms combining DEAS-based signature and independent prognostic clinical variables to predict OS and PFS in LUAD patients. **A** Nomogram of OS combining the OS signature and three clinical variables of LUAD patients. **B**–**D** Calibration curves of the nomogram at 1, 2, and 3 years. **E** Nomogram of PFS combining the PFS signature and two clinical variables of LUAD patients. **F**–**H** Calibration curves of the nomogram at 1, 2, and 3 years

Following that, on the basis of the independent prognostic factors, two novel nomograms were created for predicting OS (Fig. 7A) and PFS (Fig. 7E). The C-indices for the OS and PFS nomograms were 0.704 (95% CI 0.661–0.747) and 0.69 (95% CI 0.647–0.733), accordingly. The nomograms' significant calibration suggested that the outcomes predicted by the nomograms showed consistency with the observed data (Fig. 7B–D, F–H).

### Potential regulatory network between SFs and AS events

**Fig. 8:**
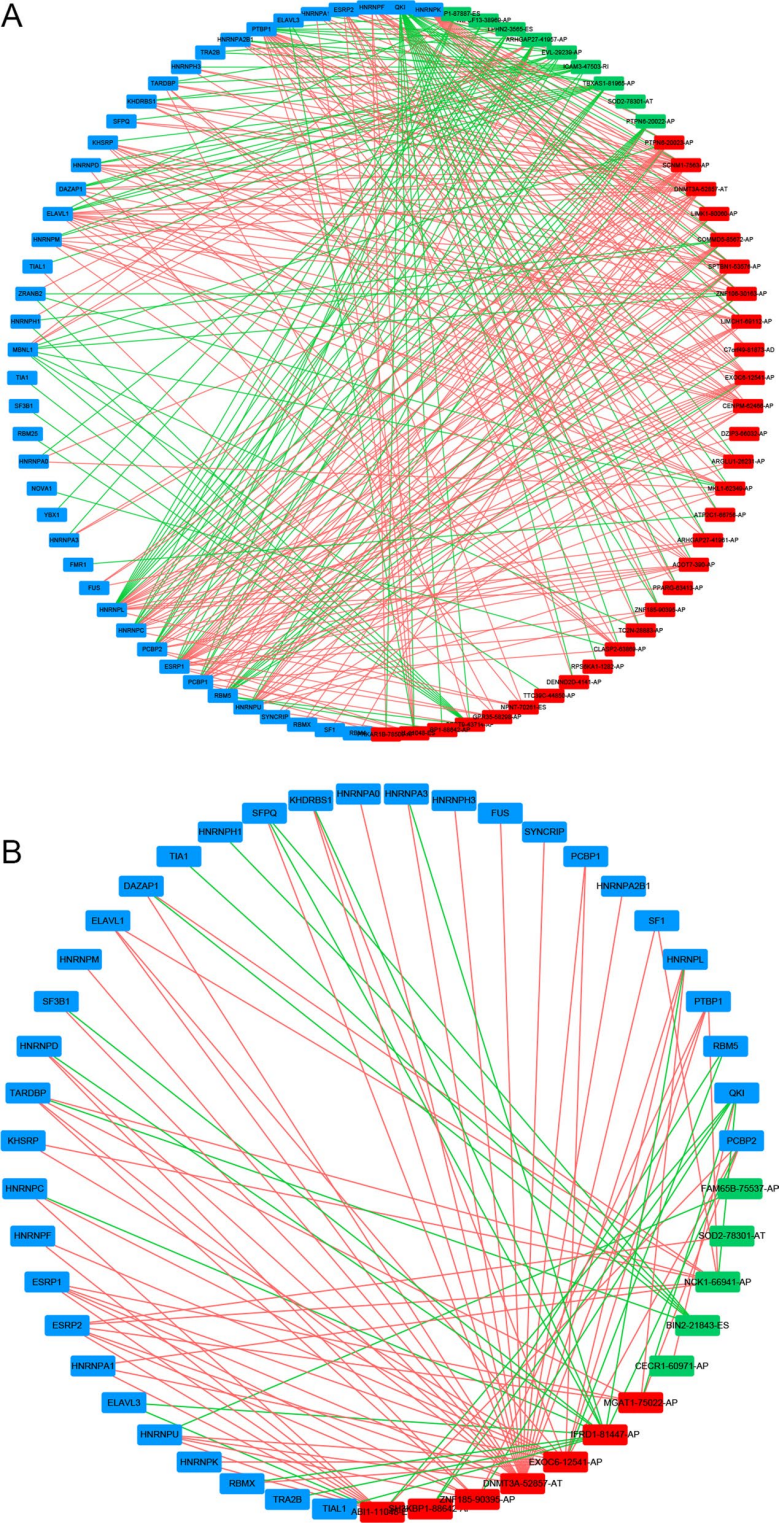
Regulatory network between SFs and AS events in LUAD. **A** OS, **B** PFS

SFs are protein factors involved in the splicing process of RNA precursors and are closely related to AS events. We retrieved 71 SFs from the SpliceAid2 databank to investigate the fundamental regulatory network between SFs and AS events in LUAD cases. We identified 42 SFs (blue) that were substantially connected to 39 AS events linked with survival, comprised of 9 favorable AS events (green) and 30 adverse AS events (red) with OS signature using Spearman correlation analyses (Fig. 8A). The PFS signature was screened out of 35 SFs (blue) that were substantially connected to 11 survival-associated AS events, with 6 adverse AS events (red) and 5 favorable AS events (green) (Fig. 8B). Many SFs were found to be associated with numerous AS events and to play opposing roles in regulating various AS events. Different SFs may also regulate a certain AS event. The underlined phenomenon elucidates that a single transcript can result in many splicing events.

### Analyzes of SOD2-78,301-AT Regulating Relationship

**Fig. 9:**
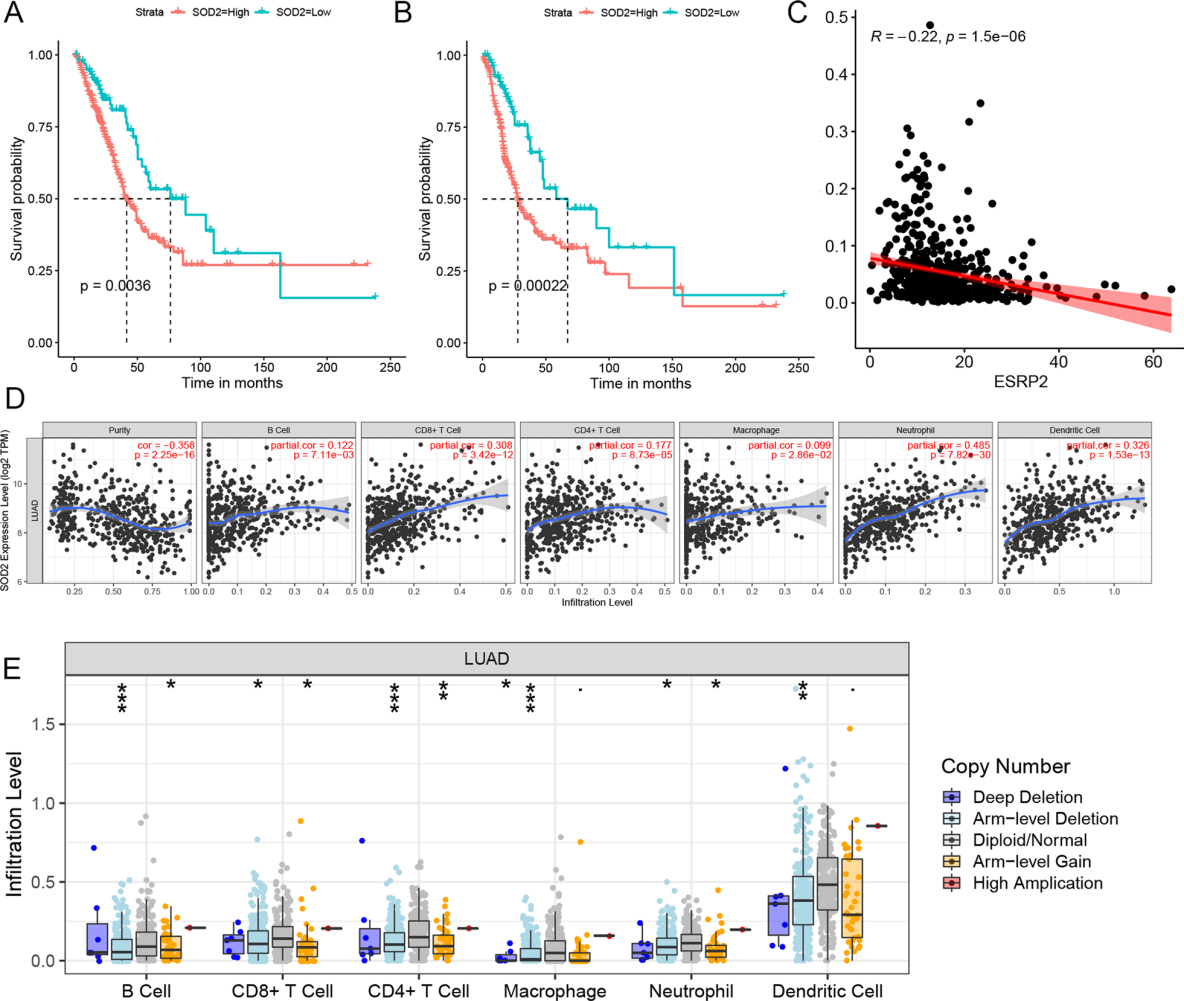
Analysis of the crucial SOD2-78,301-AT DEAS event. The K–M survival curves for OS (**A**) and PFS (**B**) of high and low SOD2-78,301-AT expression level groups. **C** The relationship between SOD2|78,301|AT and ESRP2. **D** The correlation between SOD2 expression in LUAD and abundance of immune infiltrates. **E** The correlation between somatic copy number alterations (SCAN) and abundance of immune infiltrates of SOD2

In the multivariate CRA, SOD2-78,301-AT was evaluated to be an independent OS- and PFS-associated AS and was added into both signatures as a DEAS. The expression of SOD2-78,301-A T and the prognosis of LUAD patients were also investigated. A low level of SOD2-78,301-AT was associated with considerably superior OS (Fig. 9A) and PFS (Fig. 9B), according to K–M survival curves. The link between SOD2|78,301|AT and its only SF (ESRP2) was also investigated, and the findings verified their negative regulatory interaction (Fig. 9C). Finally, in LUAD, we investigated the link between SOD2|78,301|AT's parental gene SOD2 and immune cells. There was a statistically considerable association between SOD2 expression in LUAD and the presence of immunological infiltrates (CD4 + T cells, B cells, macrophages, CD8 + T cells, neutrophils, and dendritic cells) (P < 0.05) (Fig. 9D). GISTIC 2.0 defines somatic copy number alterations (SCANs) as arm-level deletion, deep deletion, arm-level gain, diploid/normal, and strong amplification. In LUAD, box plots were used to indicate the distributions of all immune subcategories at each copy number status with SOD2 (Fig. 9E).

## Discussion

Immunotherapy has revolutionized the treatment of cancers over recent decades and possesses a considerable role in LUAD treatment. A randomized Phase III trial comparing atezolizumab with docetaxel in cases with earlier treated progressive NSCLC, which revealed that atezolizumab outperformed docetaxel in terms of OS. Across programmed death-ligand 1 and histological subcategories, atezolizumab was found to improve the survival rate. Patients who received atezolizumab had fewer treatment-associated adverse events of grade 3 or 4 than those who received docetaxel [37]. Durvalumab indicated clinically substantial enhancements in OS and PFS than the standard of care in strongly pretreated patients with metastatic NSCLC. Durvalumab + tremelimumab indicated numerical enhancements in OS and PFS relative to standard of care [38]. Immunotherapy has given people with LC novel hope, but it is not appropriate for all of them. To enhance the prognosis of LC patients, more research is required to identify immune-associated prognostic markers and to develop innovative therapeutic options. The immune-associated DEAS events were employed to build a model of risk score that accurately predicted the outcomes of LUAD patients in our investigation.

Herein, the ESTIMATE algorithm was employed to determine scores (immune as well as stromal) of LUAD generated from the TCGA database from the microenvironment's perspective. We then used K–M curves to predict the prognosis of LUAD and indicated that cases with a greater immune/stromal score exhibited a higher chance of survival. We also evaluated immune/stromal score-associated DEASs and then chose the optimal DEASs linked with the survival using LASSO CRA by comparing the profiles of transcriptional expression within LUAD cases with higher against lower immune/stromal scores. In addition, the final prognostic signature was developed, demonstrating that it is capable of accurate prediction. The LRG, in particular, had a higher chance of surviving than the HRG. Furthermore, the risk score can be used for predicting LUAD patient survival as an independent factor. This characteristic, when taken as a whole, holds a lot of promise for predicting LUAD patients' survival.

The biological functions, such as adherens junction, cell–matrix adhesion, lysosome, and others, were found to be closely linked with the development, growth, and progression of tumors, based on GO and KEGG enrichment analysis; Adherens junctions are significantly linked with the invasive and migratory potential of cancerous cells [39], for instance, E-cadherin is one of the key constituents of adherens junction, and a tumor suppressor and its loss is linked with a bad prognosis in a wide spectrum of cancers, including prostate cancer [40] and neck and head cancer [41]. Cell–matrix adhesion has also been linked to the progression of many cancers [42–44]. Lysosomes considerably contribute to the cell's degradation, and defects in them can lead to unregulated cell proliferation [45]. All of the above shows that the biological effects related to AS events are inseparable from the occurrence and development of tumors.

We also performed an analysis of unsupervised cluster to divide the TCGA LUAD cohort into three subgroups. The patients with a good prognosis had the greatest immune, stromal, and ESTIMATE scores, as well as the greatest level of immune cell infiltration, according to our findings. Immune cells are key prognostic factors in patients with LUAD in earlier research [46]. For instance, the increase in several targetable immune checkpoint molecules is linked to EMT and the microenvironment of inflammatory tumors [47]. Öjlert et al. discovered that a higher immune score and great estimations of numerous adaptive immune cell types were linked to higher PFS in LUAD patients [23]. Jones et al. discovered that the immunological signatures of cytotoxic lymphocytes and T-cell trafficking were linked to the prognosis of female malignancies like LUAD, showing that immune cell infiltration in the TME influenced treatment and survival results in LUAD cases [48].

In the current study, we also evaluated the prognostic value of DEASs. An OS-prognostic signature based on 5 DEASs (MKL1|62,349|AP, ICAM3|47,503|RI, SOD2|78,301|AT, CLASP2|63,869|AP, and EXOC6|12,541|AP) and PFS-prognostic signature based on 6 DEASs (DNMT3A|52,857|AT, NCK1|66,941|AP, SOD2|78,301|AT, IFRD1|81,447|AP, SH3KBP1|88,642|AP, and ABI1|11,048|ES) was constructed. Interestingly, among the splicing events, one overlapping AS event (SOD2|78,301|AT) was discovered with substantial variations based on OS and PFS concurrently, implying that SOD2|78,301|AT is the most plausible independent prognostic factor. Its parental gene SOD2 is a crucial enzyme for scavenging ROS generated by mitochondria, which is necessary for cellular homeostasis [49]. Reported studies have been revealed that SOD2 has been linked with the energy metabolism of colorectal cancer [50], oxidative stress in endocrine cancer [51], and stem cell reprogramming in breast cancer [52]. Other parental genes in the two signatures, in addition to this one, have been identified to perform a considerable task in the existence and progression of many cancers to differing degrees, as well as the prognosis of cancer patients. An elevated expression of MKL1 indicates an unfavorable prognosis in papillary thyroid cancer patients and enhances nodal metastases [53]. CLASP2 has been linked to the EMT and early progression of bladder cancer [54] and has also been shown to predict bladder cancer prognosis [55]. In several malignancies, DNMT3A has been shown to influence cellular apoptosis [56], cisplatin resistance [57], tumor growth, and metastasis [58]. NCK1-AS1 enhances the expression of NCK1 to aid carcinogenesis and chemo-resistance in ovarian cancer, according to Chang et al. [59]. Elevated expression of IFRD1 indicates a low survival rate in patients suffering from human colon cancers, according to Lewis et al. [60]. ABI1 has been linked to the EMT induction in prostate carcinogenesis [61], ovarian cancer metastasis [62], and neuroblastoma development, invasion, and metastasis in several investigations [63]. These risk variables were nearly all linked to cancer patients’ prognosis, even though there is little connected study on LUAD, which will require more research in the future.

In summary, a cluster model and two prognostic nomogram models which were well established to predict the survival (OS and PFS) of LUAD patients from different angles. Two prognostic nomogram models, which combine risk score models and clinical variables, allow clinicians to estimate the OS and PFS of each LUAD patient by entering the score of each parameter. However, unsupervised clustering can analyze data samples based on the inherent characteristics and find a natural grouping among the data [64, 65]. The unsupervised consensus analysis based on DEAS identified three discernable clusters with different prognosis, and significantly associated with immune and stromal scores (as shown in the figure). This implies that we can roughly estimate the prognosis of patients based on the level of tumor immune infiltration to verify and supplement the results of nomogram.Furthermore, it was found that splicing-derived new epitopes expand the potential of the immunotherapy target space [66]. The study of Peng et al. also showed that abnormal splicing events contribute to tumor progression through the influence of immune response [67], which suggests that we should consider splicing events and immunity together to evaluate the prognosis of patients.

There is no denying that our study still has significant flaws. First and foremost, an independent external validation cohort to confirm the performed DEAS-based predictive risk score signature would be preferable. Secondly, some experimental validation would have been beneficial to further confirm our data. These are also our future research directions, and we will verify these results in advanced research settings.

In conclusion, our study developed a risk score model on the basis of 11 prognostic DEASs for predicting survival rate in LUAD patients. Notably, this study also shed light on the complexity as well as the diversity of the immune microenvironment in LUAD patients that might explore the lack of therapeutic success in these individuals.

## Conclusion

Our research showed for the first time that AS events were closely related to tumorigenesis and the immune microenvironment in LUAD. We constructed two prediction models (OS and PFS) based on survival-related AS events. A total of 11 AS events are included in the models, 5 for OS and 6 for PFS, each of which is an important risk predictor. These findings provide new evidence for the interpretation of tumorigenesis and new ideas for tumor treatment.

## Supplementary Information

**Additional file 1: Fig 1.** The flowchart of the present study.

**Additional file 2: Fig 2.** **A** The K–M survival curves for PFS of high and low immune scores groups. **B** The K–M survival curves for PFS of high and low stromal scores groups. **C** The upset plot of the intersection DEAS events. **D** K–M survival curves for PFS of three clusters.

**Additional file 3.** **File 1.** Highly expressed DEAS events in the immune group.

**Additional file 4.** **File 2.** DEAS events with low expression in the immune group.

**Additional file 5.** **File 3.** Highly expressed DEAS events in the stromal group.

**Additional file 6.** **File 4.** DEAS events with low expression in the stromal group.

**Additional file 7.** **File 5.** The cluster of LUAD patient.

**Additional file 8.** **File 6.** Multivariate Cox analyses to assess independent OS prognostic variables.

**Additional file 9.** **File 7.** Multivariate Cox analyses to assess independent PFS prognostic variables.

## Data Availability

All data generated or analyzed during this study are included in the article and its Additional files 1, 2, 3, 4, 5, 6, 7, 8, 9.
