# Supplementary figures and images for: The pattern of alternative splicing in lung adenocarcinoma shows novel events correlated with tumorigenesis and immune microenvironment
Source: BMC Pulm Med. 2021 Dec 6;21:400. doi: 10.1186/s12890-021-01776-0 (PMC8647402; doi:10.1186/s12890-021-01776-0)

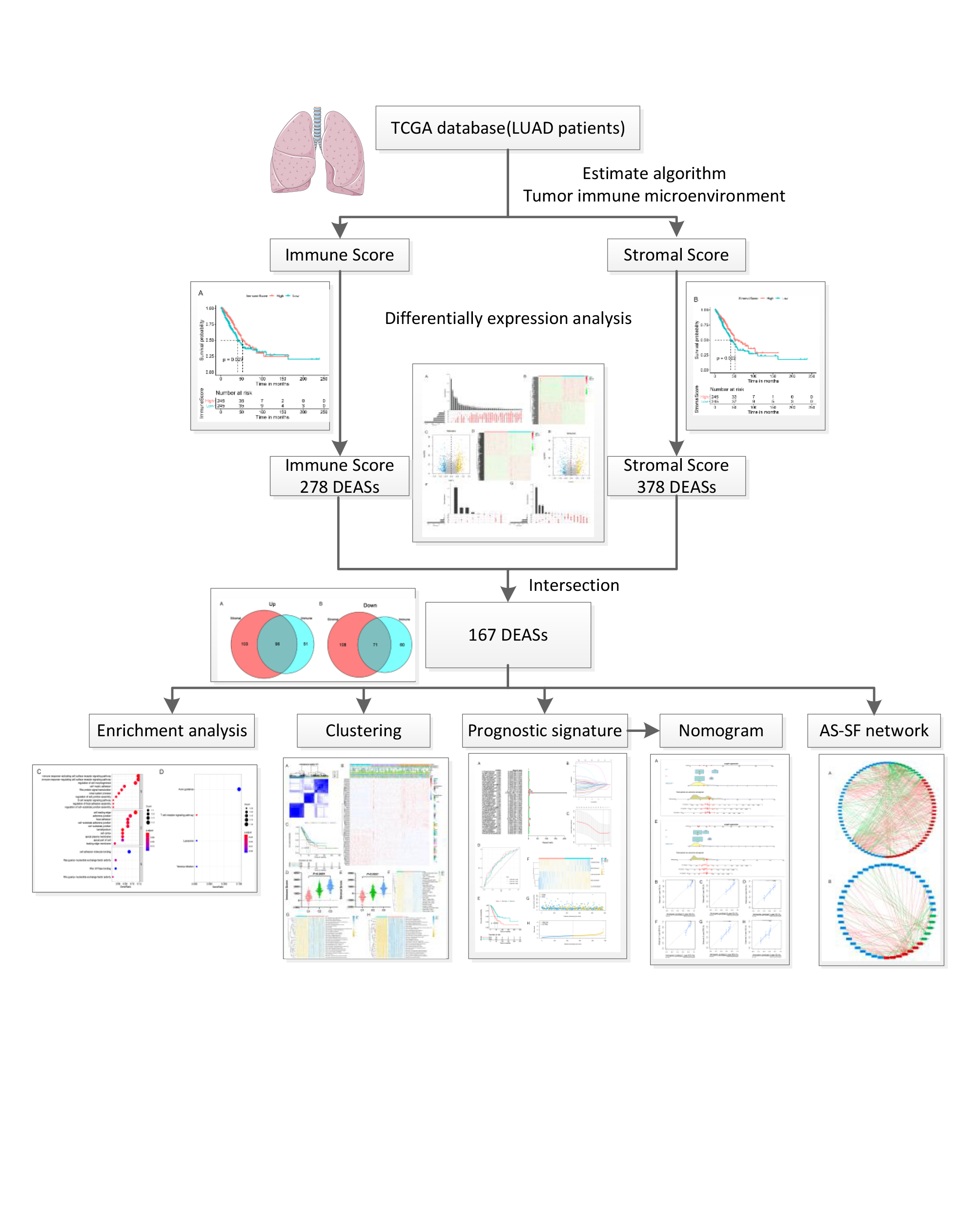

Supplement: Supplementary file 1 — Additional file 1: Fig 1. The flowchart of the present study. [file 12890_2021_1776_MOESM1_ESM.tif]

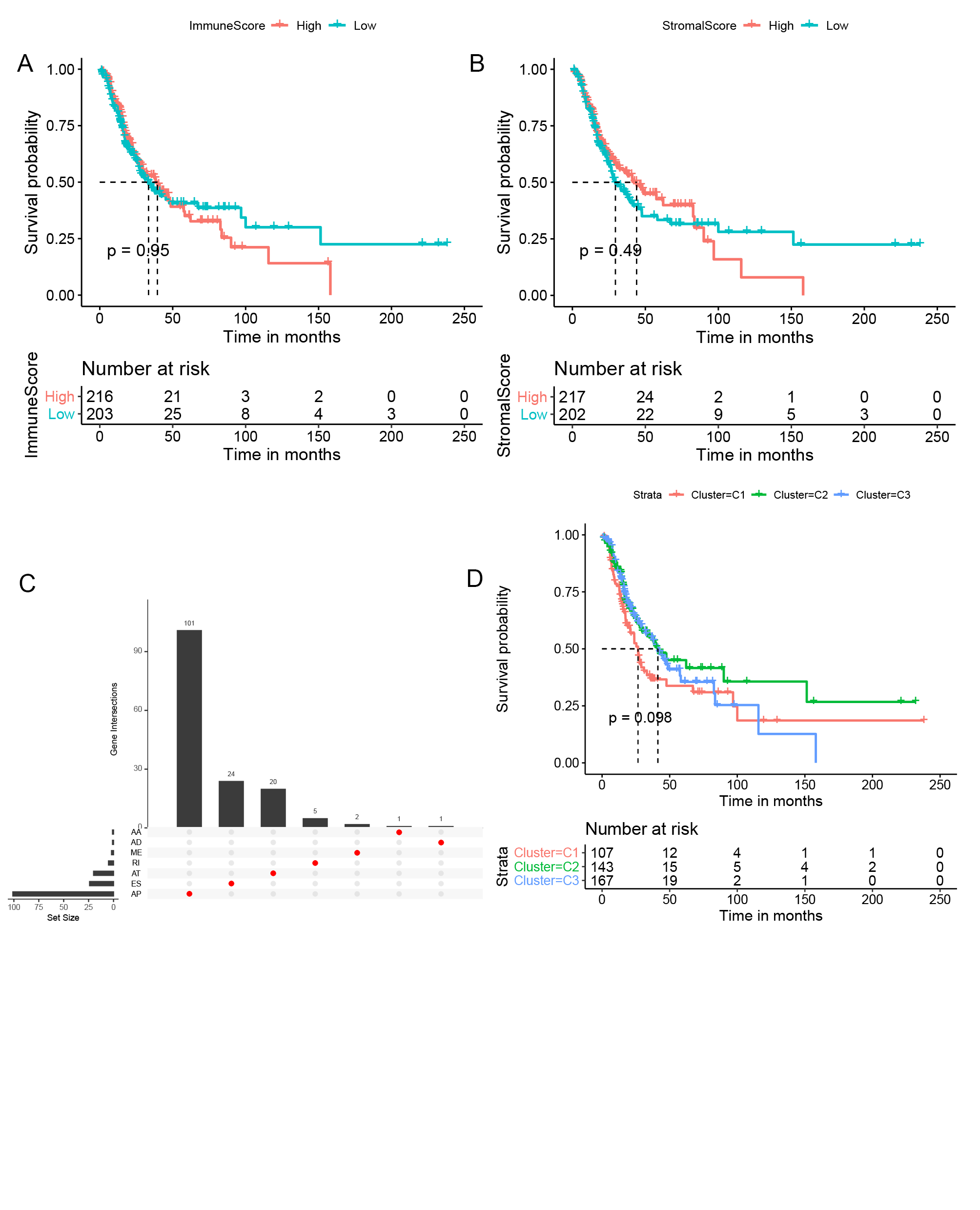

Supplement: Supplementary file 2 — Additional file 2: Fig 2. A The K–M survival curves for PFS of high and low immune scores groups. B The K–M survival curves for PFS of high and low stromal scores groups. C The upset plot of the intersection DEAS events. D K–M survival curves for PFS of three clusters. [file 12890_2021_1776_MOESM2_ESM.tif]
