# Supplementary material for: The pattern of alternative splicing in lung adenocarcinoma shows novel events correlated with tumorigenesis and immune microenvironment
Source: BMC Pulm Med. 2021 Dec 6;21:400. doi: 10.1186/s12890-021-01776-0 (PMC8647402; doi:10.1186/s12890-021-01776-0)
Supplement: Supplementary file 8 — Additional file 8. File 6. Multivariate Cox analyses to assess independent OS prognostic variables. [file 12890_2021_1776_MOESM8_ESM.docx]

Supplement file 6：multivariate Cox analyses to assess independent OS prognostic variables

| id | HR | HR.95L | HR.95H | pvalue |
| --- | --- | --- | --- | --- |
| riskScore | 1.680282133 | 1.3641949 | 2.069607536 | 1.06E-06 |
| AJCC III-IV | 1.432464118 | 0.862386463 | 2.379389681 | 0.16509758 |
| T T3-4 | 1.667692844 | 1.020570719 | 2.725141306 | 0.041227821 |
| N N123 | 1.985331746 | 1.325390342 | 2.97387269 | 0.000879854 |
| M M1 | 1.423288876 | 0.739460237 | 2.739499874 | 0.290734594 |
