# Supplementary material for: The pattern of alternative splicing in lung adenocarcinoma shows novel events correlated with tumorigenesis and immune microenvironment
Source: BMC Pulm Med. 2021 Dec 6;21:400. doi: 10.1186/s12890-021-01776-0 (PMC8647402; doi:10.1186/s12890-021-01776-0)
Supplement: Supplementary file 9 — Additional file 9. File 7. Multivariate Cox analyses to assess independent PFS prognostic variables. [file 12890_2021_1776_MOESM9_ESM.docx]

Supplement file 7：multivariate Cox analyses to assess independent PFS prognostic variables

| id | HR | HR.95L | HR.95H | pvalue |
| --- | --- | --- | --- | --- |
| riskScore | 1.491898852 | 1.312164193 | 1.696252799 | 1.01E-09 |
| AJCC III-IV | 0.957768525 | 0.612963374 | 1.496534028 | 0.849706646 |
| T T3-4 | 2.054650117 | 1.299041443 | 3.249770918 | 0.002081162 |
| N N123 | 1.619591815 | 1.132685816 | 2.315803386 | 0.008220667 |
